# Different Catechol-O-Methyl Transferase Inhibitors in Parkinson's Disease: A Bayesian Network Meta-Analysis

**DOI:** 10.3389/fneur.2021.707723

**Published:** 2021-09-24

**Authors:** Zhaoming Song, Jie Zhang, Tao Xue, Yanbo Yang, Da Wu, Zhouqing Chen, Wanchun You, Zhong Wang

**Affiliations:** ^1^Department of Neurosurgery and Brain and Nerve Research Laboratory, The First Affiliated Hospital of Soochow University, Suzhou, China; ^2^Department of Neurosurgery, Yixing People's Hospital, Yixing, China

**Keywords:** Parkinson's disease, caffeic acid O-methyltransferase, network meta–analysis, safety, efficacy

## Abstract

Parkinson's disease (PD) is a common, chronic, progressive, debilitating neurodegenerative disease. The current levodopa treatment requires the addition of other drugs, such as catechol-O-methyl transferase (COMT) inhibitors, to alleviate motor fluctuations in advanced PD. Therefore, a theoretical reference for treatment is urgently needed. In this study, an appropriate search strategy was used to screen eligible studies on different drugs to treat patients with PD from the Embase, PubMed, and Cochrane Library. The publication dates were from January 1990 to June 2021. We integrated eligible randomized controlled trials, and statistical analysis was performed on three kinds of effectiveness outcomes and two types of safety outcomes. We assessed the average difference or odds ratio between each drug and placebo and summarized them as the average and 95% confidence interval (CI), respectively. In terms of efficacy, entacapone (mean difference [MD], 0.64 h; 95% CI, 0.29–1.0), opicapone (MD, 0.92 h; 95% CI, 0.35–1.5), and tolcapone (MD, 3.2 h; 95% CI, 2.1–4.2) increased patients' total ON-time compared to placebo. Tolcapone (MD, −100 mg; 95% CI −160 to −45) reduced the total daily dose of levodopa therapy. None of these three drugs was found to have statistical significance in mean change from baseline in UPDRS part III scores when compared with others. In terms of safety, tolcapone (MD, 3.8; 95% CI, 2.1–6.8), opicapone (MD, 3.7; 95% CI, 2–7.2), and entacapone (MD, 2.2; 95% CI, 1.5–3.3) increased the number of cases of dyskinesia compared to placebo. Entacapone (MD, 1.7; 95% CI, 1.3–2.2) and tolcapone (MD, 4.3; 95% CI, 1.3–15) were more likely to cause adverse events than placebo. In conclusion, opicapone showed higher efficiency and fewer safety problems in five indicators we selected when compared with the other two drugs.

## Introduction

Parkinson's disease (PD) is a common chronic, progressive, debilitating neurodegenerative disease. It is mainly caused by the decrease of dopamine in the brain (1). PD is characterized by muscle rigidity, resting tremor, motor symptoms, and balance disturbances. Increasing evidence has reported that 2–3% of the population aged ≥65 years suffer from PD (2).

Currently, levodopa is the most effective drug in PD treatment. However, long-term use of levodopa in patients with advanced PD is often associated with end-of-dose motor fluctuations, such as dyskinesia and fluctuations (3, 4). The motor fluctuations include ON-time, in which the symptoms are controlled and the patient can move smoothly, and OFF-time, in which motor symptoms reappear or worsen (5). It is currently believed that the OFF-time is related to the weakened effect of dopaminergic drugs, which can be alleviated by maintaining a stable drug plasma level (6). Therefore, dose grading, extended-release formulations, or drug infusion pumps can be used to provide more physiological continuous dopaminergic stimulation.

Currently, many drugs are used to treat PD effectively (7). Many studies have shown that catechol-O-methyl transferase (COMT) inhibitors, such as entacapone, opicapone, and tolcapone, can prolong the plasma elimination half-life of levodopa and increase its bioavailability and effectiveness (7, 8). This is referred to as add-on, adjuvant, or adjunct therapy in PD and is a common first-line strategy for the management of PD (9). In recent years, three main clusters of COMT inhibitors were selected to treat PD—tolcapone, entacapone, and opicapone. COMT inhibitors are glucuronidated in the liver and mediate the metabolism of levodopa to 3-O-methyldopa, increasing the area under the plasma concentration-time curve of levodopa and significantly delaying the Cmax and Tmax of levodopa (10). Tolcapone is generally considered more effective than entacapone, but its clinical application is limited by the risk of liver disease and requirement of continuous liver function monitoring; therefore, it is suitable for patients who are unresponsive or intolerant to other COMT inhibitors. Entacapone is often used clinically as an adjuvant therapy for PD (11).

Research has shown that opicapone, a new type of third-generation and long-acting COMT inhibitor, can significantly reduce COMT activity and increase systemic exposure to levodopa (12, 13). Therefore, there is a need for a COMT inhibitor that is more effective and easier to use in routine clinical practice. A network meta-analysis should be performed to provide a theoretical reference for patients with PD.

## Materials and Methods

### Literature Search

In this study, an appropriate search strategy was utilized to screen eligible studies on different drugs to treat patients with PD from the Embase, PubMed, and Cochrane Library. The publication dates were from January 1990 to June 2021. The following keyword queries were used: “Parkinson's disease” OR “PD” AND “Opicapone” OR “Entacapone” OR “Tolcapone” OR “placebo” OR “levodopa.”

### Inclusion and Exclusion Criteria

The studies were selected based on the following criteria: (1) randomized controlled trials (RCTs) involving patients with advanced PD receiving levodopa; (2) each article must contain at least one outcome variable, such as the number of adverse events, number of dyskinesia symptoms, change in total ON-time, Unified PD Rating Scale (UPDRS) part III scores, and total daily dose of levodopa; (3) each article must include at least one adjuvant drug for the treatment of PD, including opicapone, entacapone, and tolcapone; (4) all subjects must be Parkinson's patients with fluctuating symptoms.

The publications were excluded according to the following criteria: (1) incomplete data or lack of statistical analysis; (2) reviews, comments, and letters; (3) duplicate articles or multiple surveys based on the same data; and 4) open-label studies;

### Quality Assessment and Data Extraction

The Cochrane Collaboration risk of bias assessment tool was used to evaluate the quality of all selected articles (14). After extracting and identifying eligible articles, two reviewers extracted relevant data for independent evaluation, including data on first author, publication year, study area, follow-up time, total number of included participants, population age, and sex ratio. In addition, if there were any disagreements during data extraction and quality assessment, a conclusion was reached after discussion with a third reviewer.

### Statistical Analysis

According to the Bayesian framework, Review Manager 5.4.1 and R4.0.3 software was used for routine pairwise meta-analysis and network meta-analysis. The mean difference (MD) and corresponding 95% confidence interval (CI) were used as valid indicators for this analysis. The chi-square q test and *I*^2^ statistic were used to evaluate heterogeneity between trials. The random effects model was used if *p* < 0.05 or *I*^2^ > 50% showed significant heterogeneity, while the fixed effects model was used if *p* > 0.05, and *I*^2^ <50% showed significant heterogeneity. The data included direct comparisons and indirect comparisons, and the results are expressed in forest plots. To analyze consistency, we compared the inconsistencies between direct and indirect sources of evidence. We compared the goodness of fit between the consistency and inconsistency models and calculated the difference between the direct and indirect estimates for one of the three comparisons in each closed loop formed by the three processes partial evaluation, all of which are compared with each other (15, 16).

In addition, a ranking curve was used to evaluate the ranking probability of each clinical outcome. A higher rank probability value indicates a more desirable attribute relative to a certain endpoint. We estimated the probability of each drug ranking for each outcome. Using the ranking probability, the processing level was summarized and reported as a surface under the cumulative ranking curve (SUCRA). The larger the SUCRA value, the better the rank of treatment outcomes.

## Results

### Study Characteristics

**Figure 1:**
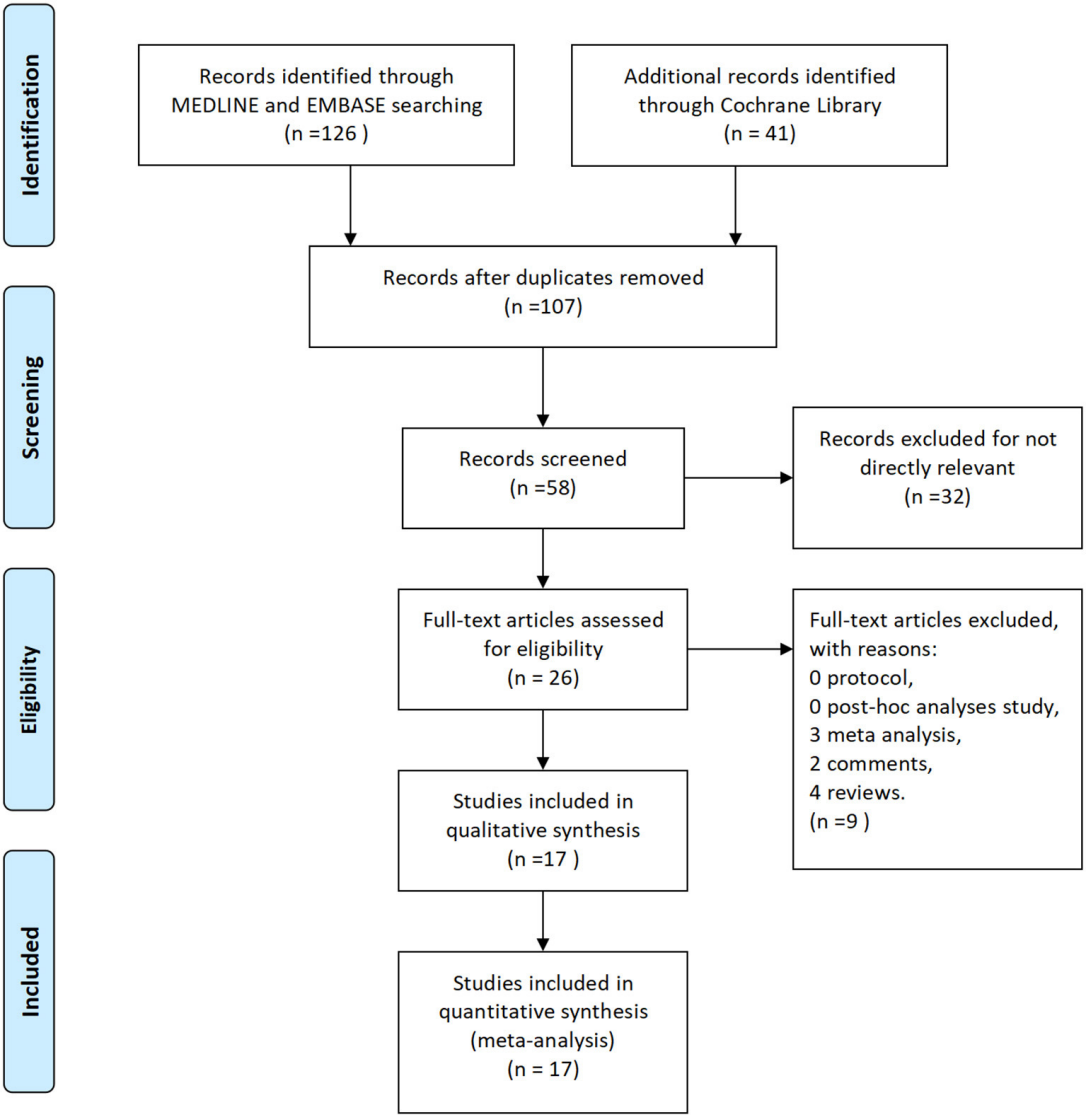
Flow diagram for study identification.

A total of 167 studies were retrieved from a preliminary literature search according to the search for related keywords. After excluding 60 duplicate studies, 107 studies remained. After a review of titles and abstracts, 81 articles were excluded because they did not meet the inclusion criteria. Thus, only 26 studies were included in the network meta-analysis. Subsequently, after excluding eight irrelevant articles, including reviews, case reports, reviews, editorials, animal studies, and basic experiments (including three meta-analyses, two comments, and four reviews), 17 eligible articles were finally included in meta-analysis. We finally selected nine articles on entacapone (17–25), three articles on opicapone (26–28), and five articles on tolcapone (29–33). A detailed flow chart of literature screening is shown in Figure 1.

The characteristics of the included studies are listed in Table 1. Specifically, 17 eligible randomized controlled trials, with a total of 4,124 patients, were included in this network meta-analysis. Among these 4,124 patients, 407 patients treated with opicapone 50 mg, 1,391 patients treated with entacapone 200 mg, and 276 patients treated with tolcapone 200 mg. The year of publication varied between 1990 and 2021. The average age of the participants included in all studies was 63.9 years, and there were more male participants than female participants. The follow-up time in all the included studies was >6 weeks.

**Table 1:** Characteristics of the included studies and outcome events.

**References**	**Countries**	**Publications**	**Treatment group (No. of participants)**	**Daily levodopa dose, mg (mean ± SD)**	**Male (%)**	**Mean age ± SD (year)**	**Study period**	**Outcome events**
Dupont et al. (29)	7	Movement disorders	PLA (33) vs. TOL (32)	PLA 588.2 ± 33Tol 665.9 ± 36.7	PLA 57.6% TOL 53.1%	PLA 66 ± 8TOL 66 ± 9	6 weeks	b,c,d
Waters et al. (34)	Canada, US	Neurology	PLA (102) vs. TOL (98)	PLA 364.3 ± 13.2TOL 381.9 ±13.2	PLA 59.8% TOL 60.2%	PLA 67 ± 10TOL 63 ± 11	24–48 weeks	b,c,e,
Rajput et al. (35)	Canada, US	Neurology	PLA (66) vs. TOL (67)	PLA 948 ± 46.9TOL 865.8 ± 47.4	PLA 71% TOL 78%	PLA 65 ± 10TOL 64 ± 9	12 weeks	b,c,e
Rinne et al. (17)	16	Neurology	PLA (86) vs. ENT (85)	PLA 705 ± 283ENT 701 ± 293	PLA 55.2% ENT 54.6%	PLA 62.8 ± 8.7ENT 63.7 ± 9.88	24 weeks	a,b
Baas et al. (33)	Europe	J Neurol Neurosurgery Psychiatry	PLA (58) vs. TOL (59)	PLA 660.9 ± 46.6TOL675.8 ± 42.4	PLA 60% TOL 56%	PLA 64 ± 8TOL 63 ± 9	12 weeks	a,c,e
Shan et al. (31)	China	Can. J. Neurol. Sci.	PLA (20) vs. TOL (20)	PLA 930 ± 131.6TOL 795 ± 71.3	PLA 80% TOL 85%	PLA 67 ± 7TOL 63 ± 4	6 weeks	a,b,c
Poewe et al. (19)	Germany, Austria	Acta Neurol Scand	PLA (104) vs. ENT (197)	PLA 572 ± 329ENT 570 ± 273	PLA 48% ENT 40%	PLA 61.1 ± 9.9ENT 60.7 ± 9.6	24 weeks	a,b,c,d,e
Brooks and Sagar (18)	UK, Republic of Ireland	J Neurol Neurosurg Psychiatry	PLA (57) vs. ENT (115)	PLA 712 ± 369ENT 682 ± 390	PLA 40% ENT 60%	PLA 64.7 ± 8.5ENT 65.9 ± 8.9	24 weeks	a,b,c,d,e
Fénelon et al. (20)	UK	J Neural Transm	PLA (63) vs. ENT (99)	N/A	PLA 60% ENT 64%	PLA 65.0 ± 6.61ENT 63.5 ± 9.96	12 weeks	a,c,d,e
Reichmann et al. (25)	N/A	Acta Neurol Scand	PLA (96) vs. ENT (174)	PLA 533 ± 231ENT 566 ± 243	PLA 59% ENT 54%	PLA 66 ± 9ENT 67 ± 8	13 weeks	a,c,d,e
Rascol et al. (24)	Europe, Israel, Argentina	Lancet	PLA (229) vs. ENT (227)	PLA 697 ± 295ENT 706 ± 321	PLA 58% ENT 61	PLA 64.8 ± 8.8ENT 63.0 ± 9.4	18 weeks	d,e
Mizuno et al. (22)	Japan	Movement disorders	PLA (95) vs. ENT (88)	PLA 431.1 ± 130.7ENT 455.1 ± 161.0	PLA 44.2% ENT 46.6%	PLA 62.7 ± 9.9ENT 62.7 ± 8.7	8 weeks	a,b,d,e
Ferreira et al. (21)	Europe, South America	CNS Neuroscience & Therapeutics	PLA (49) vs. ENT (50)	PLA 712 ± 298ENT 709 ± 307	PLA 65.3% ENT 54%	PLA 64.1 ± 10.01ENT 65.3 ± 8.6	8 weeks	d
Rascol et al. (23)	20	Clinical Neuropharmacology	PLA (247) vs. ENT (234)	N/A	PLA 60% ENT 58%	PLA 63.6 ± 8.82ENT 63.7 ± 9.88	18 weeks	a
Ferreira et al. (36)	20	Lancet Neurol	PLA (121) vs. ENT (122) vs. OPI (115)	PLA 675 ± 302ENT 645 ± 323OPI 695 ± 338	PLA 59% ENT 62% OPI 60%	PLA 64.3 ± 9.3ENT 63.7 ± 7.7OPI 63.5 ± 9.2	14–15 weeks	a,c,d,e
Lees et al. (27)	12	JAMA Neurology	PLA (135) vs. OPI (147)	PLA 714 ± 338OPI 700 ± 312	PLA 52.6% OPI 60.5%	PLA 61.5 ± 8.9OPI 65.5 ± 8.4	14–15weeks	a,b,d,e
Takeda et al. (28)	Japan	Movement disorders	PLA (147) vs. OPI (145)	PLA 422.3 ± 170.1OPI 445.3 ± 175.8	PLA 56% OPI 60%	PLA 68.5 ± 8.6OPI 67.4 ± 7.8	14–15 weeks	a,b,d,e

*PLA, Placebo; OPI, Opicapone; ENT, Entacapone; TOL, Tolcapone; N/A, not application; a, the total ON-time; b, the scores of UPDRS PartIII; c, the total daily dose of levodopa; d, adverse event; e, dyskinesia symptom*.

Supplementary Figure 1 shows the established networks for comparison. In Supplementary Figure 1, each node represents a treatment. Connections between nodes denote direct comparisons; the node size and thickness of connections vary according to the number of studies involved in a comparison.

### Quality Assessments of the Selected Literature

**Figure 2:**
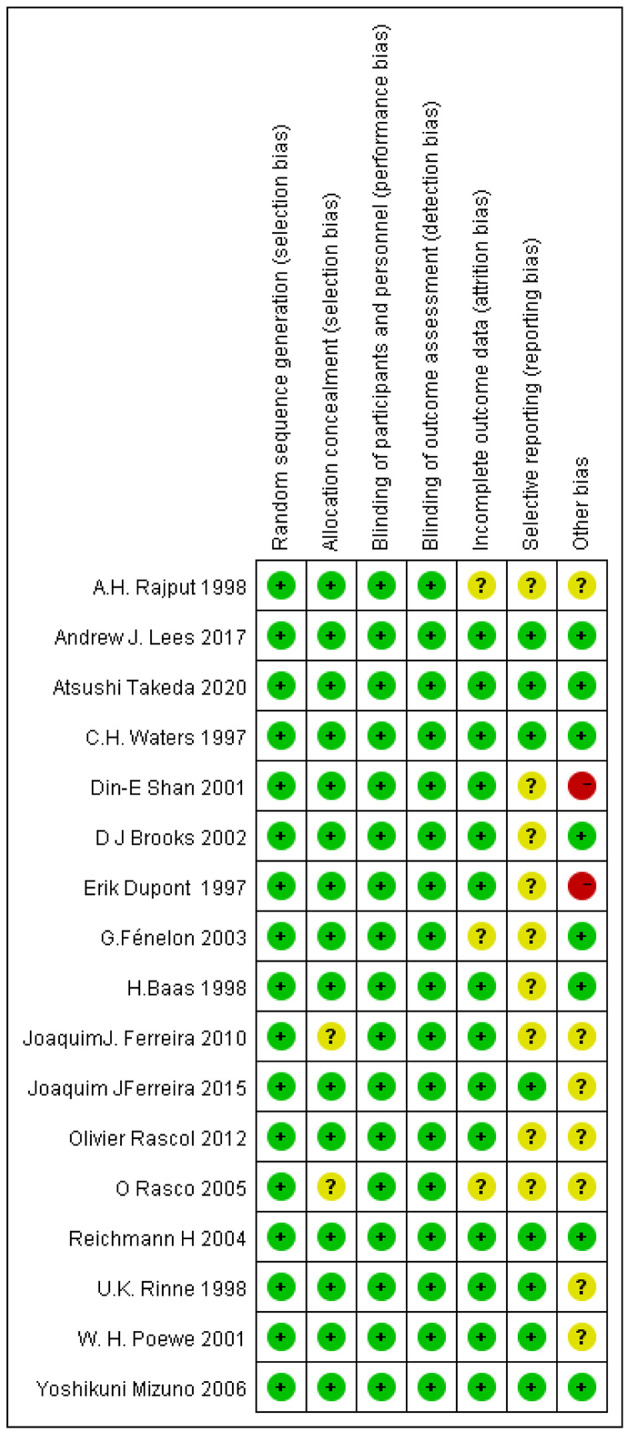
Risks of bias assessment.

The quality evaluation of randomized controlled trials (RCTs) revealed that the overall quality of publications we included was relatively high as exhibited in Figure 2. Despite the assessment results of many articles show unclear in terms of selective reporting indicator, there were only two literatures having higher risks in terms of other bias which was caused by insufficient follow-up of 6 weeks.

### Network Meta-Analysis

**Figure 3:**
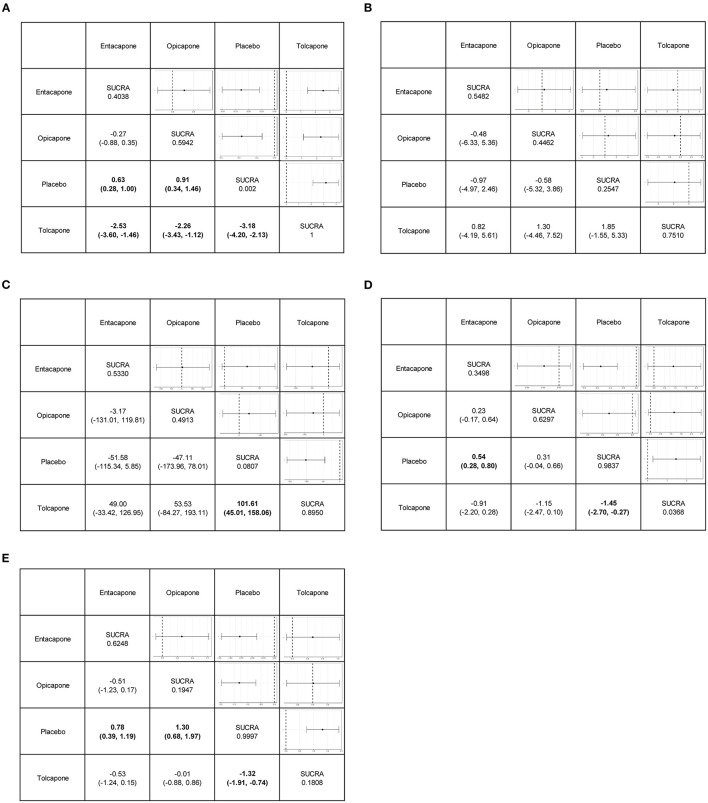
League tables for the outcomes of safety and efficacy generated using the random effects model. **(A)** Change in total ON-time. **(B)** Change in UPDRS part-III (motor) score. **(C)** Total daily dose of levodopa. **(D)** Any adverse events. **(E)** Dyskinesia.

We performed network meta-analysis to explore the differences between different drugs used to treat patients with PD. In terms of efficacy, entacapone (MD, 0.64 h; 95% CI, 0.29–1.0), opicapone (MD, 0.92 h; 95% CI, 0.35–1.5), and tolcapone (MD, 3.2 h; 95% CI, 2.1–4.2) increased patients' total ON-time compared to placebo. Tolcapone (MD, −100 mg; 95% CI, −160 to −45) reduced the total daily dose of levodopa therapy compared to placebo. However, there three drugs were no statistically significant differences in the mean change from baseline in UPDRS part III scores (Figure 3).

In terms of safety, tolcapone (MD, 3.8; 95% CI, 2.1–6.8), opicapone (MD, 3.7; 95% CI, 2–7.2), and entacapone (MD, 2.2; 95% CI, 1.5–3.3) increased the number of cases of dyskinesia compared to placebo. Entacapone (MD, 1.7; 95% CI, 1.3–2.2) and tolcapone (MD, 4.3; 95% CI, 1.3–15) were more likely to cause adverse events than placebo. Opicapone was not significantly associated with the probability of causing adverse events compared to placebo (Figure 3).

### Rank Probability

**Figure 4:**
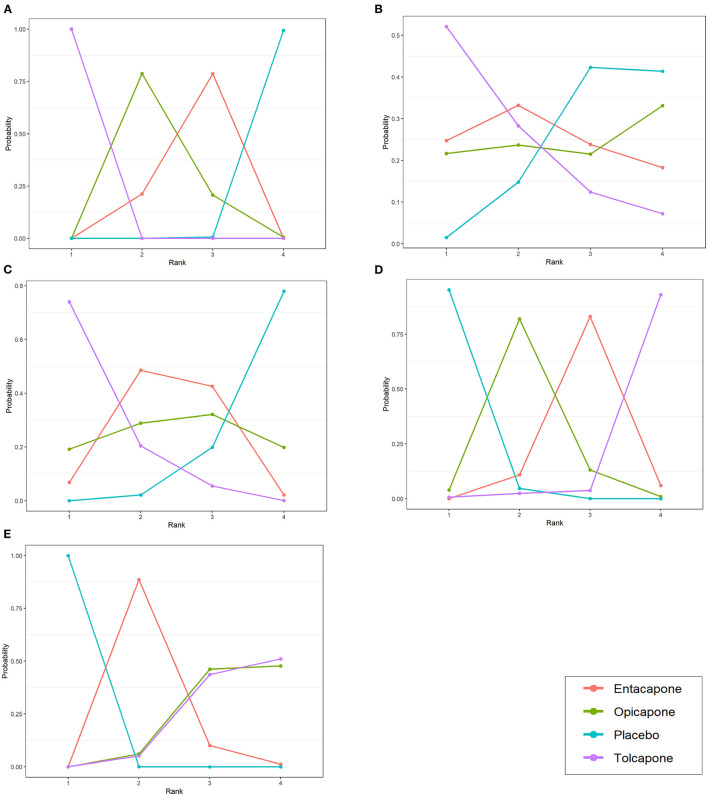
Probability ranks for outcomes of the safety and efficacy generated using the random effects model. **(A)** Change in total ON-time. **(B)** Change in UPDRS part-III (motor) score during the on state. **(C)** Total daily dose of levodopa. **(D)** Any adverse events. **(E)** Dyskinesia. The positive results favors mean that drugs with higher rank have better effect or higher safety in treatment.

Figure 4 shows a ranking chart of the probability of each target strategy ranked in terms of efficiency. According to the SUCRA values and rank probability of the efficacy of the three drugs, tolcapone (SUCRA, 1.00) ranked the highest in extending the ON-time, followed by opicapone (SUCRA, 0.5942) and entacapone (SUCRA, 0.4038). Further, tolcapone (SUCRA, 0.895) ranked the highest in reducing the total daily dose of levodopa therapy, followed by entacapone (SUCRA, 0.533) and opicapone (SUCRA, 0.4913). In addition, tolcapone (SUCRA, 0.0368) ranked the highest in the occurrence of adverse events, followed by entacapone (SUCRA, 0.3498) and opicapone (SUCRA, 0.6297). Lastly, tolcapone (SUCRA, 0.1808) ranked the highest in the occurrence of dyskinesia, followed by opicapone (SUCRA, 0.1947) and entacapone (SUCRA, 0.6248). For these two safety indicators, the smaller the SUCRA value, the worse the safety and the higher the probability of adverse events.

### Heterogeneity and Consistency Analysis

**Figure 5:**
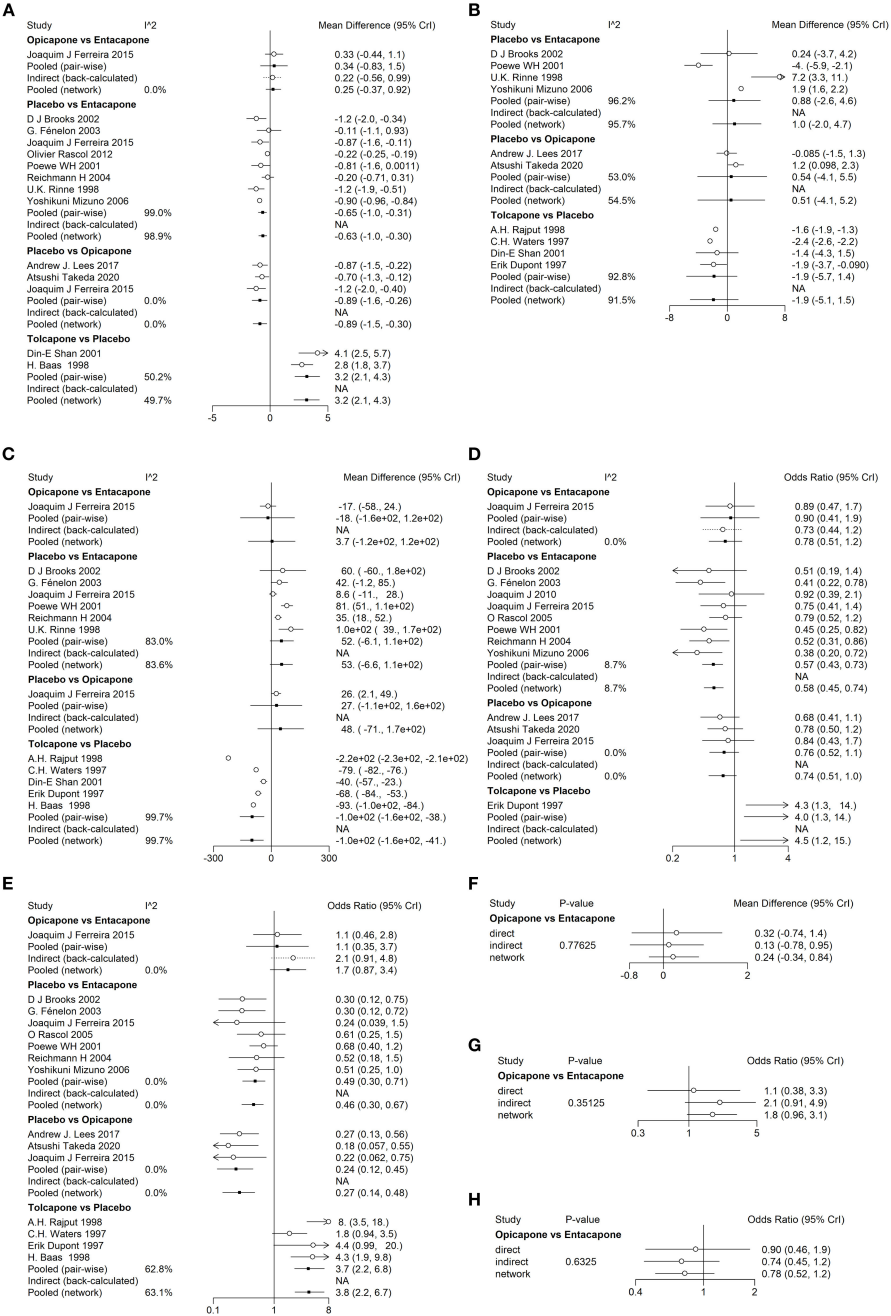
Forest plots for the heterogeneity and consistency of efficiency and safety indicators. The forest plots of heterogeneity show in **(A–E)**. **(A)** Change in total ON-time. **(B)** Change in UPDRS part-III (motor) score during the on state. **(C)** Total daily dose of levodopa. **(D)** Any adverse events. **(E)** Dyskinesia. The forest plots of consistency shows in **(F–H)**. **(F)** Change in total ON-time. **(G)** Dyskinesia. **(H)** Any adverse events.

To assess the heterogeneity among the included studies, we performed heterogeneity analysis on each indicator we chose which compared the difference between direct and indirect comparisons. The pairwise *I*^2^ values of different indicators was show in Figures 5A–E. According to *I*^2^ values of overall networks, we chose to use random effect model to perform meta-analysis in networks of change in total ON-time (*I*^2^ = 98.29317%), total daily dose of levodopa (*I*^2^ = 99.25013%) and change in UPDRS part III (motor) scores (*I*^2^ = 93.57806%). The other two indicators, the number of dyskinesia symptom (*I*^2^ = 1.792398%) and any adverse events (*I*^2^ = 0%), were meta-analyzed by fixed effect model as was stated in methods. To find out the consistency of three networks containing indirect comparisons, we used the node-splitting model to test the differences between direct and indirect comparisons. The goal was to determine the consistency between direct and indirect evidences of a particular node (split node). We also found no obvious inconsistencies in the network model with indirect sources which results were shown in Figures 5F–J. Therefore, the results of the consistency model were reliable. In addition, the potential scale reduction factor value of all parameters was limited to 1, showing good convergence and effectiveness.

## Discussion

To the best of our knowledge, this is the first network meta-analysis to compare the safety and efficacy of common COMT inhibitors as an adjunct therapy for advanced PD. Previous studies have suggested that entacapone, opicapone, and tolcapone can alleviate the symptoms of PD (10, 37). However, several factors hamper the use of currently available COMT inhibitors, that is, the moderate efficacy and multiple dosing of entacapone and the risk of liver toxicity with tolcapone. Opicapone, a new long-acting, peripherally selective COMT inhibitor, has been proven to have a good effect on PD treatment (38). In addition, there are no data on the comparison between COMT inhibitors to determine which drug has a better effect and lower risk to patients with advanced PD. In this study, we chose three efficiency and two safety indicators to evaluate the difference between entacapone, opicapone, and tolcapone treatments in 4,124 patients with advanced PD. Among these three drugs, tolcapone had the best efficiency and worst safety for patients. Opicapone and entacapone had a considerable therapeutic effect that was slightly inferior to that of tolcapone, while opicapone showed the lowest risk in all safety indicators.

The three chosen efficiency indicators included ON-time, UPDRS part III scores, and reduced daily levodopa dose. The ON-time of patients, as a complement to the ON-time, was changed most by tolcapone, which is consistent with the findings of a previous study (39). Opicapone ranks second in improvement of ON-time, while entacapone ranks the last, but has statistical significance compared to placebo. As a novel COMT inhibitor, opicapone has been proven to prolong ON-time when patients switch to it from entacapone (36, 39). In terms of reducing levodopa dose, tolcapone had the best effect on advanced PD, while that of entacapone and opicapone showed no statistical significance compared with placebo. This outcome was greatly influenced by the low inclusion of these three drugs. Although tolcapone exhibited significant statistical differences, the initial levodopa doses taken by patients in the included RCTs were different. The initial levodopa dose taken before tolcapone treatment ranged from 65.8 ± 47.4 (35) to 381.9 ± 13.2 (34), which may lead to the incredibility of its statistical significance. More clinical studies are required to enrich the data and categorically analyze the change in daily levodopa dose under different initial dose conditions. Lastly, we used the UPDRS part III as an efficiency indicator, which is the most widely used PD scale and recognized as the most comprehensive, effective, and reliable scale (40). However, none of the three drugs compared achieved statistical significance. This result may be explained by the fact that we included patients with advanced PD, but the UPDRS part III has the highest sensitivity in the early stages of PD (41). Our study and previous RCTs and meta-analyses have shown similar outcomes, which suggest that this scale may not be appropriate for rating the effects of drugs in advanced PD.

The number of patients with any adverse event and the number of patients with dyskinesia were chosen as the two indicators to evaluate safety. In our analysis, opicapone had the lowest adverse event rates, while tolcapone had the highest rate. Considering our efficiency results, tolcapone exhibits the highest efficiency, but at the expense of safety. Previous studies have revealed serious liver toxicity associated with tolcapone use. Tolcapone is a drug that easily passes through the blood–brain barrier and has a moderate systemic clearance rate. It is almost completely metabolized, mainly forming glucuronate metabolites. Tolcapone binds to plasma proteins in the body and causes severe liver damage (42). While opicapone had the lowest adverse event rate, it had the highest prevalence rate of dyskinesia among these three drugs. This suggests that the adverse events of opicapone mainly include dyskinesia. Despite having a medium rate of these two indicators, entacapone had the lowest efficiency in changing the ON-time and had similar rates of other indicators to those of opicapone. Its bioavailability is low, and the time to inhibit COMT is short, which reduces its effectiveness (43). Opicapone does not easily pass through the blood–brain barrier, has high bioavailability, and has a long effective time. Therefore, it has a high potential for the treatment of PD.

However, our analysis has some limitations. (1) The number of patients included in the tolcapone group was small; therefore, our results may only reflect the impact of small studies. (2) Only one study reported reduced total daily dose of levodopa in the opicapone group. Therefore, the outcome has a certain deviation, and further research is needed. (3) Our data and conclusions are based on statistical analysis. The clinical validity of this method is unclear, and further research is needed. (4) The UPDRS part III scores were not statistically significant, which may be due to the small sample size in some articles. (5) The study period of some articles is <8 weeks. Short-term studies may reduce the credibility of the conclusion.

In conclusion, our network meta-analysis of three types of COMT inhibitors, including opicapone, compared the advantages and limitations of different drugs through statistical analysis of 17 RCTs. Compared with other two drugs, tolcapone had better effectiveness, but worse safety. Entacapone had intermediate effectiveness and safety. Opicapone as an adjuvant therapy for PD prolonged the total ON-time of patients with PD and was less prone to adverse events; thus, based on a comprehensive assessment of all indicators we analyzed, it was considered to be better than that of the other two drugs. This finding provided a theoretical reference for clinical treatment of Parkinson's disease. Due to the limitations we stated before, this conclusion remains to be examined by more clinical researches in the future.

## Data Availability Statement

The original contributions presented in the study are included in the article/Supplementary Material, further inquiries can be directed to the corresponding author/s.

## Author Contributions

ZW and WY were the principal investigators. ZS and TX designed the study and developed the analysis plan. JZ analyzed the data and performed the meta-analysis. ZC and YY contributed to the writing of the article. DW and ZS revised the manuscript and polished the language. All authors read and approved the final submitted paper.

## Funding

This work was supported by the Suzhou Health Talents Training Project (GSWS2019002) and National Natural Science Foundation of China (81771256).

## Conflict of Interest

The authors declare that the research was conducted in the absence of any commercial or financial relationships that could be construed as a potential conflict of interest.

## Publisher's Note

All claims expressed in this article are solely those of the authors and do not necessarily represent those of their affiliated organizations, or those of the publisher, the editors and the reviewers. Any product that may be evaluated in this article, or claim that may be made by its manufacturer, is not guaranteed or endorsed by the publisher.

## Abbreviations

COMT: catechol-O-methyl transferase
RCT: randomized controlled trial
RR: risk ratio
SUCRA: surface under the cumulative ranking curve
CI: confidence interval
MD: mean deviation.
