# Supplementary material for: Different Catechol-O-Methyl Transferase Inhibitors in Parkinson's Disease: A Bayesian Network Meta-Analysis
Source: Front Neurol. 2021 Sep 24;12:707723. doi: 10.3389/fneur.2021.707723 (PMC8497751; doi:10.3389/fneur.2021.707723)
Supplement: Supplementary file 1 [file Image_1.pdf]

Title: Different COMT Inhibitors in Parkinson's disease: A Bayesian network meta-analysis

Zhaoming Song[1,#], Jie Zhang[1,#], Tao Xue[1], Yanbo Yang[1], Da Wu[2], Zhouqing Chen[1], Wanchun You [1,*], Zhong Wang [1,*]

[1] *Department of Neurosurgery & Brain and Nerve Research Laboratory, The First Affiliated Hospital of Soochow University, Suzhou, Jiangsu Province, 215006, China*

[2] *Department of Neurosurgery, Yixing People's Hospital, Yixing, 214200, China.*

Supplement I

Network comparison of five indictors. A: Change in total ON-time; B: Change in UPDRS partIII (motor) score; C: Total daily dose of levodopa; D:Any adverse events; E: Dyskinesia

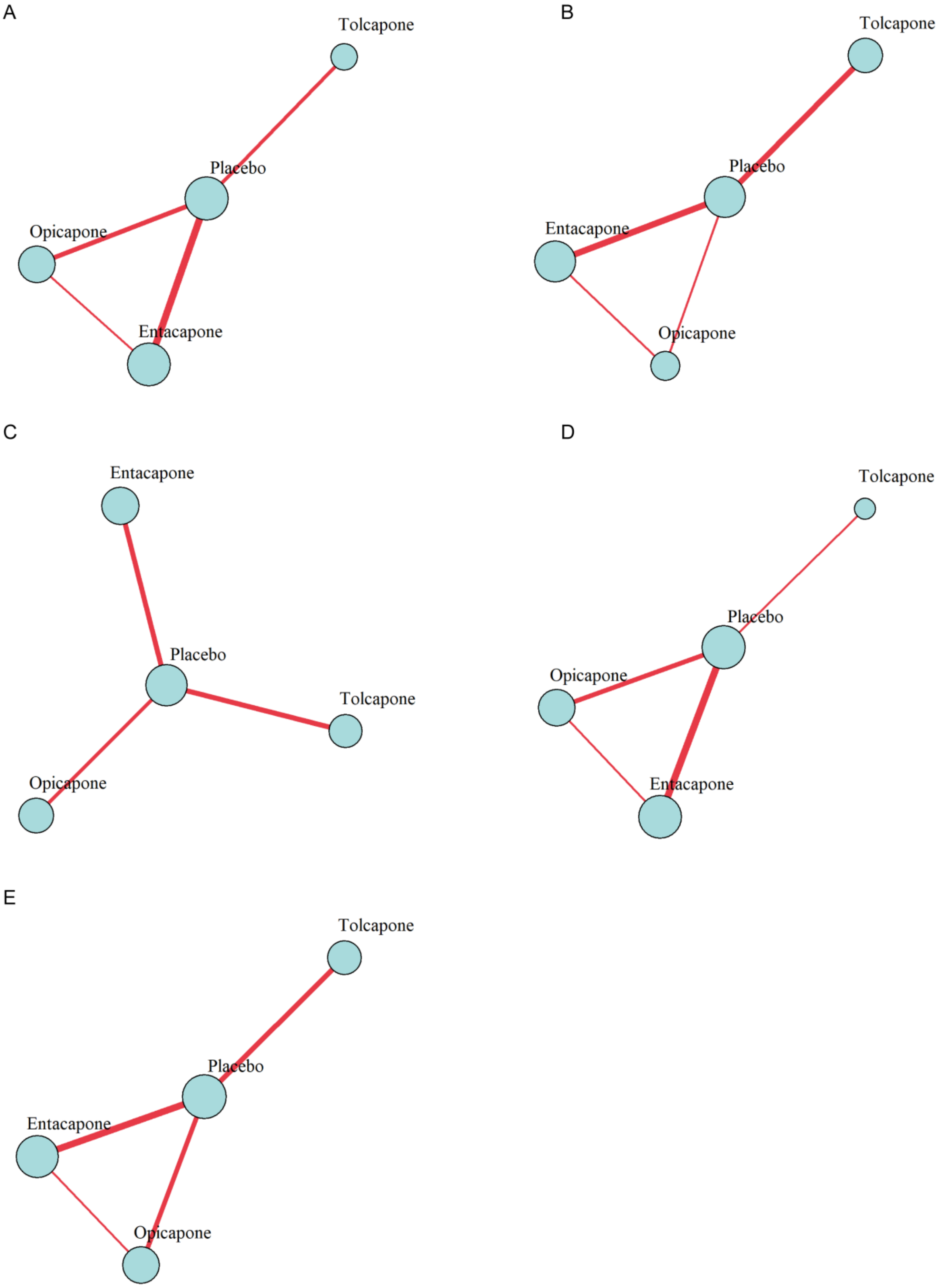

S1.Network comparison of five indictors. A: Change in total ON-time; B: Change in UPDRS partIII (motor) score; C: Total daily dose of levodopa; D:Any adverse events; E: Dyskinesia
